# Surgical Cases Treated at the Buffalo General Hospital

**Published:** 1870-12

**Authors:** C. C. F. Gay


ART. VI.—*Surgical Cases treated at the Buffalo General Hospital.* By C. C. F. GAY, M. D.

*Condylomata.*—I. D., aged 19 years, admitted to the Hospital on August 18th.

He has a condylomatous growth located at the anus, which has required two years to attain its present size. It will measure four inches in its longest diameter, two inches in its transverse diameter, and is at about the same thickness, being adherent at a broad base. It is very vascular and sensitive to the touch. Defecation is difficult and extremely painful.

August 18th I removed this growth with the knife. The operation was attended by considerable hemorrhage. There were found, after removal of the tumor, small warty excrescences within the border of the anus, which were removed with the scissors. Equal parts of chromic acid and water was applied to the surface, and in five weeks the wound was healed with no indications of a return of the disease.

*Retention of Urine caused by Traumatic Stricture.*—Mr. D., aged 23 years, admitted September 19th. An injury had been inflicted upon his perineum, six weeks before his admission, by a kick from a man while the patient was lying in bed. He became unconscious, and remained so for twelve hours; from this time to the present he has had great difficulty in passing his urine, until it resulted in complete retention. A fruitless attempt had been made to catheterize him on the day before his admission. The resident physician also attempted to introduce the catheter, but did not succeed and sent for me. I found the patient in great pain, with an over-distended bladder. Chloroform was administered; and after trial of three quarters of an hour, I became convinced of the utter impossibility of entering the bladder with a catheter through the urethra. I, therefore, plunged the curved trochar into the bladder, through the rectum, and evacuated a large quantity of urine, after which I passed a small flexible catheter up through the canula, over which the canula was withdrawn, and the catheter allowed to remain *in situ*. On the next day found the patient comfortable, and urine passing through the catheter. The instrument I allowed to remain, with the view to give time for the false passage within the urethra to heal, and with the hope that the urine might be evacuated within a few days through its natural outlet.

September 21st.—Urine is passing in small quantity through the urethra. Withdrew the cather from the rectum on the 22d, since I find sufficient urine continues to pass naturally, to afford relief.

A Gouley filiform bougie was now passed through the urethra, over which the sound was guided into the bladder.

26th. Patient passes urine very comfortably in small stream, and feels well and thinks he must leave, and did leave the Hospital next day, feeling himself sufficiently relieved to go to work.

*Amputation of the Thigh, by the Circular method, below the Trochanter.*—Before the application of anæsthetics to surgery, it was a mooted question whether or no it were advisable to wait until after reaction before operating. And since the use of anæsthetics, the question has been reopened; but, doubtless the propriety or impropriety of operating before or after the patient re-acts from the shock of injury, will be left, as it should, to the discretion of the operator. The less the surgeon is hampered and restricted by rules, the better will it be probably both for patients and surgeons.

These preliminary remarks have suggested themselves in noting the only fatal case of capital operation which occurred during a continuous service at the Hospital of over seven months.

Thos. Osborne was injured by a locomotive on the Erie Rairoad. The wheels of the engine passed over the left leg and thigh, producing a compound comminuted fracture of the leg and thigh. The vessels were injured, and no means had been used to arrest hemorrhage; and he was conveyed to the Hospital, a distance of nearly two miles, while the blood was dripping from his limb through the wagon in which he was lying. I saw him within an hour after the injury. It was necessary either to cut down and ligate the femoral, to amputate or let the patient die without any attempt to dress his leg.

The limb could not be saved in any event; therefore, after having given stimulus, waiting for a short time for re-action, and then administering ether, I amputated, assisted by Dr. Miner and the house physician, by the circular method, just below the lower trochanter, Mr. Harrington, the acting house physician, compressing the femoral artery with his thumb. But very little blood was lost. The patient did not rally, but died twelve hours after the operation, from shock.

*Varicose Veins.*—During my term of service, two cases were treated for radical cure by the use of potassa cum calce.

I prefer this method to any other. It is safe, almost painless, and is successful.

The first patient treated, aged 26 years, had become debilitated from previous illness of several months. The enlarged veins were undoubtedly the result of general debility. I produced five eschars upon the left leg, and three upon the right. In four weeks he was discharged cured, since which there has been no reappearance of the disease, the veins appeared to have been obliterated.

The second patient was Benjamin Hawes, aged 61 years. He entered the Hospital with a varicose ulcer, and varicose veins, upon his left leg.

Potassa cum calce, made into a paste by alcohol, was applied directly over the enlarged veins at five points of considerable distance from each other. In twenty minutes the paste was washed off with vinegar. The eschars thus made were of the size of a pea, but before the slough came away, they had enlarged to the size of a nickel penny. Poultice of slippery elm was constantly applied.

There was no constitutional disturbance nor scarcely any local pain. At the expiration of six weeks the eschars had not only healed, but the varicose ulcer had also healed. The cure seems to be radical. When the patient is standing up, the leg looks smooth, and there exists no longer any appearance of enlarged veins.

